# Optical coherence tomography angiography for noninvasive evaluation of angiogenesis in a limb ischemia mouse model

**DOI:** 10.1038/s41598-019-42520-3

**Published:** 2019-04-12

**Authors:** Liwei Wang, Zuoguan Chen, Yongjun Li, Jing Yang, Yuejie Li

**Affiliations:** 1 Institute of Biomedical Engineering, Chinese Academy of Medical Sciences and Peking Union Medical College, Tianjin, 300192 P.R. China; 2 Department of Vascular Surgery, Beijing Hospital, National Center of Gerontology, Chinese Academy of Medical Sciences and Peking Union Medical College, Beijing, 100730 P.R. China

## Abstract

We developed an optical coherence tomography angiography technique by improving the speckle contrast algorithm and the imaging process. This technique, which can achieve angiogenesis imaging *in vivo* without increasing trauma, was used to evaluate the microvasculature in limb ischemia mice. Sixteen left hindlimb ischemia mice were randomly allocated into CuSO_4_ and saline groups. Within 7 days after treatment, limb ischemic damage, temperature and histological staining were assessed by traditional methods. In addition, angiogenesis was evaluated using an optical coherence tomography angiography system *in vivo*. All results were compared. After 7 days of treatment, both the ischemic tissue damage score and temperature ratio of the CuSO_4_ group were significantly higher than those of the control group (all P < 0.05). The number of CD31-positive endothelial cells in the CuSO_4_ group (0.1836 ± 0.0153) was significantly greater than that in the saline control group (0.0436 ± 0.0069) (P < 0.001). Optical coherence tomography angiography showed that the vessel area density of mice in the CuSO_4_ group (0.2566 ± 0.0060) was significantly greater than that of mice in the control group (0.2079 ± 0.0202) (P = 0.027). Optical coherence tomography angiography represents a practical and effective method for observing angiogenesis in the mouse hindlimb *in vivo* without increasing trauma.

## Introduction

With the development of society, problems associated with aging have become increasingly serious. In recent years, the prevalence of peripheral arterial disease (PAD) has markedly increased, affecting nearly 20% of people over 60 years of age^1,2^. Critical limb ischemia (CLI) is the most severe clinical manifestation of PAD and results in the chronic inadequate supply of oxygen and nutrients to the extremities^3^. CLI has become a major source of morbidity and mortality, which causes considerable economic and societal burdens. However, studies have shown that 10–18% of patients have limited benefits from successful macrovascular revascularization. The main reason for unsatisfactory outcomes is the lack of focus on microcirculation^4–6^. Therefore, quantitative and qualitative evaluations of microvascular networks are important in limb ischemia mouse models because they are typically used as preclinical models for the evaluation of novel therapeutic methods.

Various methods have been proposed to evaluate microcirculation; no one method is perfect. Vascular imaging techniques, such as computed tomography (CT) and magnetic resonance imaging (MRI), are not sensitive for analyzing angiogenesis in small capillaries *in vivo*^7^. Micro-CT represents an improved device that can analyze angiogenesis in small animals. However, micro-CT requires invasive instrumentation, highly qualified personnel and toxic contrast agents^8,9^. Typically, Doppler ultrasound, which is a noninvasive technique that does not require radiation exposure or contrast enhancement, is used as an outcome measurement; however, it has low resolution and is insufficient for microvascular analysis^10,11^. Currently, histological techniques, such as immunohistochemical staining of CD31 or CD34, are the standard methods of quantifying capillary vasculature^12,13^. However, histology requires the sacrifice of animals, and it is a time-consuming method due to serial sectioning. In addition, infrared thermal imaging systems and the ischemia damage score are also used as indirect outcome measurements for angiogenesis, although these methods are not objective and mainly depend on the experience of researchers. Thus, a noninvasive method that is fast and precise for evaluating angiogenesis in mouse limb muscle is very important.

Optical coherence tomography angiography (OCTA), which has been used in ophthalmology for many years, is a noninvasive technology for *in vivo* microcirculation imaging that does not require the use of an exogenous contrast agent^14,15^. In 2013, Poole K. M. *et al*. first combined hyperspectral imaging and optical coherence tomography (OCT) for the intravital acquisition of comprehensive vascular structure and functional information in the mouse limb ischemia model^16^. This approach is attractive because multiple physiological parameters can be noninvasively analyzed through the skin at all timepoints within the same animal. However, one limitation is that this imaging method cannot sufficiently monitor microvessels. Subsequently, Poole K. M. *et al*. developed a 100 kHz, 1060 nm swept-source OCT system capable of imaging over a wider area with improved imaging depth^17^. This approach has been validated on two different mouse strains, and the results established the potential for speckle variance OCT as a tool for quantitative, preclinical screening of pro- and anti-angiogenic therapies. The speckle variance method for imaging vessel morphology has several strengths, including sensitivity to flow in microvessels, angle independence, fast acquisition, and only a slight increase in computational complexity^18^. Poole K. M. *et al*. implemented noninvasive imaging through the skin of the hindlimb and obtained longitudinal measurements. However, the skin weakens the light energy, and capillaries in the skin may also disturb the image of angiogenesis in muscle tissue.

Therefore, we were inspired to implement skin-free method instead of non-invasive imaging method due to three reasons. First, the skin-free method could avoid the disturbance of the skin capillaries and become more sensitive to distinguish angiogenesis from larger vessels, which may contribute to investigating different ischemic mechanisms in the further study. The angiogenesis in the muscle deserve our more concerns. Second, it is very important to detect the same region for comparing the repeated measures at the different timepoints, and the skin-free method could contribute to this and increase the persuasion of the experiment results. Third, the skin-free method doesn’t increase the invasive procedures because the skin needs to be incised to expose the muscle during the process of establishing limb ischemia model (at day 0) and collecting muscle specimens (at endpoint day 7). That is why we use OCTA system imaging at day 0 and day 7. We developed a new speckle contrast algorithm with high signal-to-noise and anti-interference ability and redesigned the mechanical structure of our OCTA system making it more accommodate to fix the animal. According to our previous studies^19^, the Institute of Cancer Research (ICR) mouse limb ischemia model was used for evaluating angiogenesis because the genetic map of mice is rather similar to the humans. In addition, CuSO4 was used to promote angiogenesis as an experimental group because it could significantly upregulate the expression of VEGF^20^. The histological study and temperature measurements are also clearly presented to compare with the result of OCTA. The establishment of this approach may provide a better method for the quantitative, preclinical screening of angiogenic therapies and may contribute to investigating different ischemic mechanisms in limb ischemia mouse model.

## Results

### Ischemic limb damage assessment

**Figure 1:**
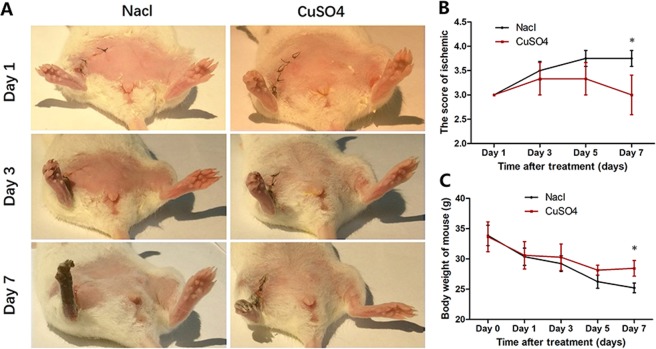
The assessment of ischemic limb damage. (**A**) representative photographs of hindlimbs on days 1, 3, and 7 after treatments. (**B**) Mouse body weight on days 0,1,3,5 and 7 after treatments. (**C**) grading the hindlimb necrosis on day 1,3,5,7 after treatments. Five grades were used to measure the degree of limb ischemic: 4 = any amputation; 3 = severe discoloration or subcutaneous tissue loss or necrosis; 2 = moderate discoloration; 1 = mild discoloration; and 0 = normal hindlimb. (*P ≤ 0.05).

The onset of ischemia was confirmed by an infrared thermal imaging system (FLIR E6 System, USA) after the femoral artery ligation surgery. Images of the mouse hindlimbs were acquired by a digital camera on days 1, 3 and 7 after treatment. Digital images of the limbs showed that the group treated with saline suffered more severe limb tissue necrosis than the CuSO_4_ group, especially at day 7 (Fig. 1A). The ischemic degree of mouse hindlimbs in each group was assessed by the 5-grade method on days 1, 3, 5 and 7 after treatment. The ischemic scores of the two groups were similar on day 1 and then increased up to 5 days after treatment. The ischemic score increased slowly in the CuSO_4_ group compared with that in the saline group on days 3 and 5. On day 7 after treatment, the ischemic tissue damage scores of the CuSO_4_ and control groups were 3.00 ± 0.75 and 3.75 ± 0.46 (P = 0.035), respectively. In the CuSO_4_ group, 25% of the animals suffered amputation (grade 4), whereas the ratio in the saline group was 75% (Fig. 1B). Mouse body weight was also recorded every two days. The body weight of mice treated with saline decreased significantly throughout the study, whereas the body weight of animals treated with CuSO_4_ decreased slowly and remained constant from day 5 to 7. On day 7, the body weights in the CuSO_4_ and saline groups were 28.44 ± 1.99 g and 25.20 ± 1.61 g, respectively (P = 0.024) (Fig. 1C).

### Temperature assessment

**Figure 2:**
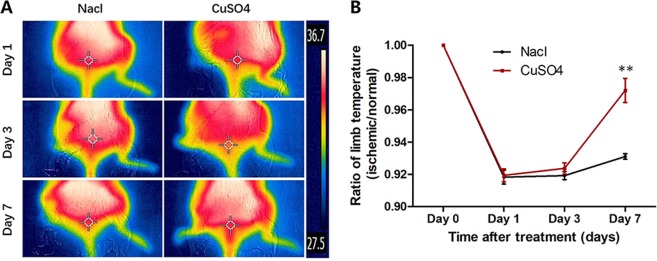
Limb temperature assessment. (**A**) Temperature obtained by FLIR on days 1, 3, and 7 after treatments. Red, strong signal; blue, weak signal. (**B**) The temperature ratio of ischemic to normal hindlimbs. (**P ≤ 0.01).

The hindlimb temperature was monitored *in vivo* by an infrared thermal imaging system at days 1, 3 and 7 after treatment (Fig. 2A). Preoperatively, the ratio of the temperature between the two hindlimbs for all animals was set to 1.0. On day 1 after treatment, the ratio of the temperature between the ischemic and normal limbs was 0.9194 ± 0.0089 in the CuSO_4_-treated group and 0.9183 ± 0.0087 in the control group. On day 3, the temperature recovered slightly in both groups of animals. However, on day 7 after treatment, the temperature clearly recovered in both groups and the temperature ratio of the mice in the CuSO_4_ group (0.9720 ± 0.0149) was significantly greater than that of the mice in the control group (0.9310 ± 0.0030) (P = 0.01) (Fig. 2B).

### Histological assessment

**Figure 3:**
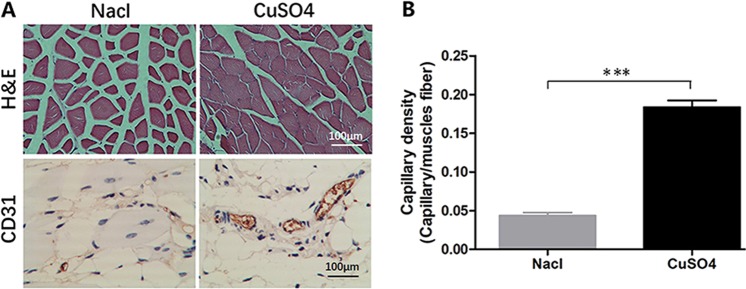
Histological analysis (x400). (**A**) Hematoxylin and eosin staining and CD31 immunohistochemical staining. (**B**) The quantify of CD31-positive endothelial cells. (***P ≤ 0.001).

Hematoxylin-eosin staining of the muscle tissue of mice on the 7th day showed that atrophy of the gastrocnemius muscle in the CuSO4 group mice was significantly less serious than that in the control group mice (Fig. 3A). Immunohistochemistry staining results on the 7th day after treatment indicated significantly larger numbers of CD31-positive endothelial cells in the CuSO4 group (0.1836 ± 0.0153) than the saline control group (0.0436 ± 0.0069) (P < 0.001) (Fig. 3A,B).

### OCTA assessment of ischemic limb

**Figure 4:**
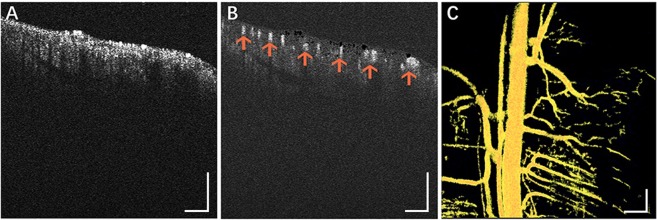
*In vivo* microcirculation imaging of a in the healthy mouse limb [5.0 mm × 5.0 mm × 2 mm]. (**A**) cross-sectional (XZ) OCT structural image; (**B**) cross-sectional vascular ISC-OCTA image; (**C**) en-face image of ISC-OCTA microvasculature. Scale bars = 500 μm.

*In vivo* microcirculation imaging of the mouse limb was demonstrated with improved speckle contrast optical coherence tomography angiography (ISC-OCTA). We imaged the vasculature of a healthy mouse limb, and en-face images of the ISC-OCTA microvasculature were acquired, as shown in Fig. 4. Figure 4A shows a cross-sectional OCT structural image that contains static tissue and dynamic blood flow signals. Figure 4B shows cross-sectional vascular ISC-OCTA images, which fully showed the presence of the limb vessels (arrows in the figure). Figure 4C shows an en-face image of the ISC-OCTA microvasculature.

**Figure 5:**
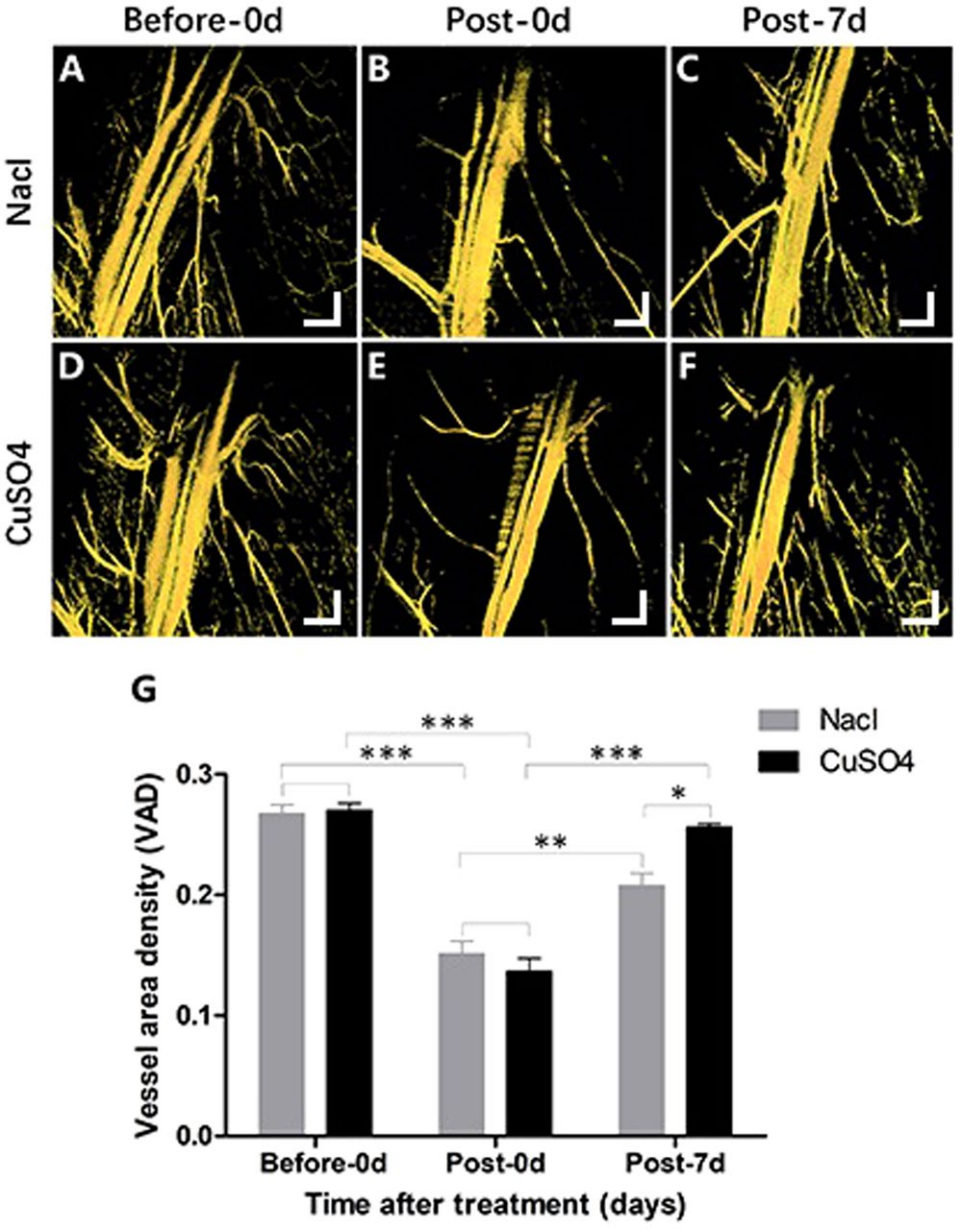
En-face images [5 mm × 5 mm] of the mouse limb vasculature. (**A**–**C**) En-face images of the mouse limb vasculature were acquired by ISC-OCTA at days 0 before, after surgery, and after 7 days of treatment in the control group; (**D**–**F**) were the ISC-OCTA images in the CuSO_4_ group. (**G**) the quantify of vessel area density. (n = 8, *P ≤ 0.05, **P ≤ 0.01, ***P ≤ 0.001). Scale bars = 500 μm.

To evaluate the effectiveness of OCTA in the limb ischemia mouse model, images of the mouse hindlimbs were acquired on day 0 before and after surgery using the OCTA system and compared with the blood flow images after 7 days of treatment (Fig. 5). The distribution of vessels in the mouse limbs was similar between the saline and CuSO_4_ groups before and after the surgery. The vessels were significantly reduced after surgery. Before surgery, the OCTA image vessel area densities (VADs) in the CuSO_4_ and control groups were 0.2703 ± 0.0118 and 0.2677 ± 0.0144 (P = 0.850), respectively. After surgery, the OCTA image VADs in the CuSO_4_ and control groups were 0.1365 ± 0.0217 and 0.1513 ± 0.0210 (P = 0.270), respectively. After 7 days of treatment, the VAD of the CuSO_4_ group was significantly greater than that of the control group, with values of 0.2566 ± 0.0060 and 0.2079 ± 0.0202, respectively (P = 0.027). The repeated measures in the CuSO_4_ (P < 0.0001) and control group (P = 0.0008) were statistical difference by ANOVA.

## Discussion

**Figure 6:**
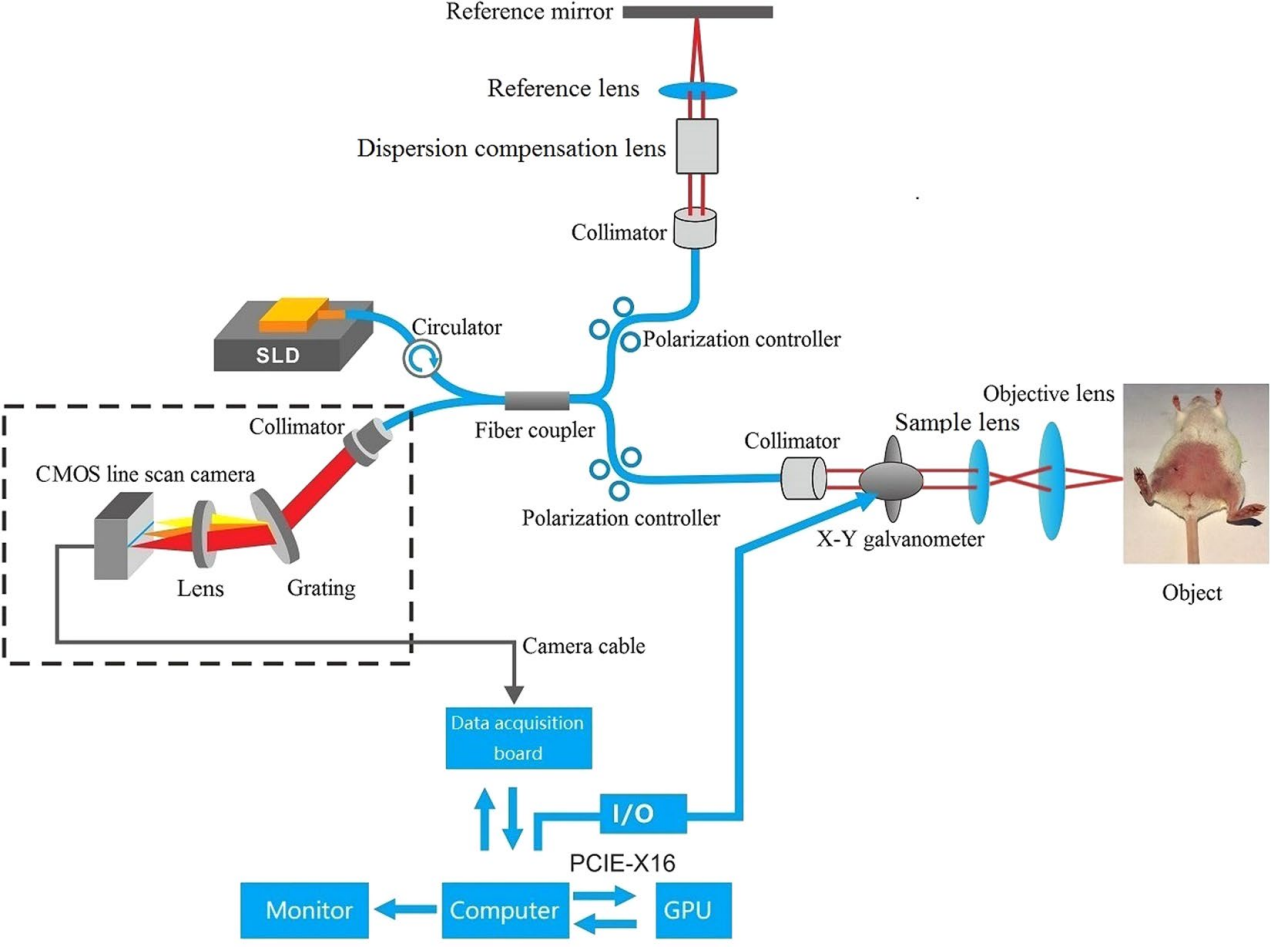
Schematic of the OCTA system. The OCTA system with a broadband super luminescent diode at 840 nm (Inphenix Inc, Livermore, CA, USA) for imaging *in vivo*, the light source has a bandwidth range of 50 nm. Lateral and axial resolution are 10 μm and 5 μm, respectively. including a polarization controller, collimator, x-y galvanometer, sample lens and objective lens in the sample arm, polarization controller, collimator, dispersion compensation lens and reference lens in the reference arm. The spectrum was collected by CMOS camera with a 2,048 pixel line scan at a rate of 70 K lines/s.

OCTA is a well-established method for noninvasive, high-resolution, repeatable and three-dimensional imaging of biological tissues^21^ and it has been used in various fields of biomedical research, especially in ophthalmology and dermatology^22,23^. Angiogenesis must be evaluated after the treatment of limb ischemia in a mouse model. As reported by Poole K. M. *et al*., the application of OCTA has dramatically increased dynamic information on blood flow in a noninvasive manner. Clearly, OCTA presents several advantages, such as noninvasive, high-resolution and convenient imaging of superficial vessels^16,17^. However, Poole K. M. *et al*. implemented noninvasive imaging through the skin of the hindlimb. To better separate the capillaries from the large blood vessel, we designed an OCTA system and improved the imaging process. First, we redesigned the structure of the OCTA system, which facilitated the fixation of the animal during imaging (Fig. 6). Second, the ISC-OCTA algorithm was clearly able to show minimally visualized angiogenesis at an axial resolution of 5 μm (Fig. 4). Third, we used the vessel area density as an indicator to quantify angiogenesis (Fig. 5). Fourth, we implemented OCTA imaging without probing through the skin to analyze muscle angiogenesis during operation (at day 0 and endpoints), which did not increase trauma. The imaging results were clearly improved because interference of the skin was avoided.

Copper is an essential element with a long history of use in humans. However, a direct role of copper in facilitating angiogenesis was shown two decades ago^24^. CuSO_4_ has been demonstrated to promote angiogenesis *in vivo*^25,26^. A study conducted by Chandan K showed the first evidence that cooper-inducible VEGF expression is sensitive to copper and that the angiogenic potential of copper can be used to accelerate ischemia improvements^27^. The study showed that CuSO_4_ (10 μM) resulted in a greater than 2.5-fold increase in the VEGF expression level compared with resting cells in standard culture. Further increases in the concentration of the CuSO_4_ treatment from 25 to 50 μM significantly increased the VEGF expression level^27^. Therefore, in our study, we injected a solution of CuSO_4_ (40 μl, 4 μg/ml) to stimulate angiogenesis in an ischemia limb model to investigate the sensitivity and effectiveness of OCTA in analyzing angiogenesis.

The microvascular signal was compared before and after femoral artery ligation and 7 days after treatment. First, the VAD results in both groups were consistent with the other results, including the number of CD31-positive endothelial cells, limb temperature and limb ischemia score. Before treatment, differences were not observed in the VAD values between the two groups. All of these results indicate that the subsequent VAD results were valid. Second, after 7 days of treatment, the number of CD31-positive endothelial cells, the recovery of limb temperature, and the ischemic tissue damage score of the CuSO4 group were significantly better than those of the control group (all P < 0.05). These results demonstrate that CuSO4 effectively stimulates angiogenesis in a limb ischemia model. Furthermore, the VAD of the CuSO4 group was more noticeable than that of the control group, and the OCTA results were consistent with the abovementioned indicators. In addition, OCTA is more convenient, fast and objective than traditional methods. All of these analytical results demonstrate that OCTA is an effective method of analyzing the microvasculature of the mouse hindlimb. Thus, the VAD could be a potential indicator for assessing limb ischemia.

Our group demonstrated that OCTA can be used to evaluate angiogenesis in ischemia limbs of mice. However, the depth of detection is limited due to the characteristics of the light source. Tissue has a great influence on spectral absorption; thus, selecting an appropriate detection spectrum is beneficial for improving imaging depth, which enables the visualization of deep tissue structures in a single scan. In addition, swept-source OCT provides uniform sensitivity over the entire scanning area, which overcomes the signal roll-off observed for spectral domain OCT. In future research, we will use a high-speed swept-source OCT system with a suitable wavelength (1060 nm/1310 nm) to increase the detection depth and improve the image quality by increasing the number of A-lines per B-scan, reducing the number of B-scans per position and improving the processing speed of the algorithm^28,29^. Based on the abovementioned improvements, we could apply OCTA to several other studies. First, we plan to compare the difference between images acquired through the mouse limb skin and not through the mouse limb skin. We believe that the skin-free imaging process may not only increase the muscle angiogenesis measuring depth but also provide more exact vascular information. An analysis of the results provided additional vascular information for deep tissues and a better vascular evaluation. Second, we used this novel method to investigate different ischemic mechanisms in a mouse limb ischemia model because it could separate large blood vessels from capillaries. Third, we further compared this approach with Doppler ultrasound and micro-CT for vascular assessment. This approach is able to utilize the advantages of both Doppler ultrasound and micro-CT and could represent a better choice for the quantitative analysis of mouse limb muscle angiogenesis.

OCTA provides a practical and effective method of observing the microvasculature of a mouse hindlimb, and it could be useful in the assessment of angiogenesis in mouse hindlimb ischemia models.

## Materials and Methods

### Imaging system

A schematic of the OCTA system is shown in Fig. 6. In this study, we developed a Fourier domain OCT (FD-OCT) system with a broadband superluminescent diode at a wavelength of 840 nm (Inphenix, Inc., Livermore, CA, USA) for *in vivo* imaging. The light source has a bandwidth range of 50 nm and provides a measured axial resolution of ~5 μm. The light divides the incident beam into a reference arm and sample arm using a 10:90 fiber coupler. In the sample arm, the sample light was coupled into a custom-made imaging probe with a pair of galvanometer scanners to achieve x-y scanning and collect the light backscattered from the sample. The optical power incident on the tissue was below 1 mW. The imaging probe includes a polarization controller, collimator, x-y galvanometer, sample lens and objective lens. By adjusting the position of the sample lens, the system provides a lateral resolution of 10 μm. In the reference arm, the light is delivered into the reference mirror through a polarization controller, collimator, dispersion compensation lens and reference lens. Dispersion compensation is mainly performed via hardware compensation and software compensation. Hardware compensation is conducted by equipping the system with a dispersion compensation lens group and a polarization controller in the reference arm. Software compensation is conducted via dispersion compensation by setting a compensation coefficient. The spectrum is collected by a complementary metal-oxide semiconductor (CMOS) camera with a 2,048-pixel line scan at a rate of 70 K lines/s. The measured sensitivity (in air) of the OCTA system is 103.10 dB at a position near zero-delay line (250 micrometer), and the sensitivity roll-off is ∼7.21 dB at a depth of 1.25 mm.

For 3-D OCT volume imaging, a typical raster scanning protocol is implemented with the system. The OCT system collects 256 A-lines in the X direction and 2,048 B-scans (256 consecutive locations, 8 frames per location) in the Y direction, and it presents a 2.0 mm range in the Z direction. The 3-D OCT volume data consist of a field of view of 5.0 mm × 5.0 mm.

### ISC-OCTA processing technique

**Figure 7:**
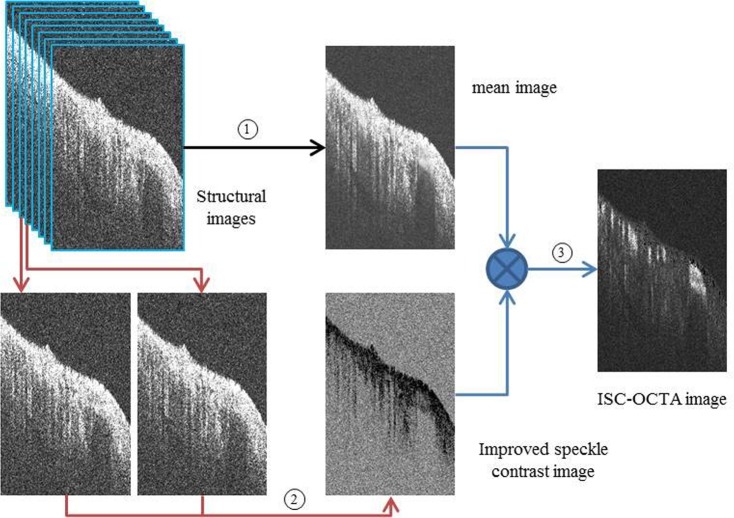
Schematic of the ISC-OCTA algorithm. 8 consecutive B-Scan frames were repeatedly captured at each transversal location, 8 consecutive frames were averaged in step 1, contrast image was acquired by performing speckle contrast analysis on two consecutive frames in step 2, ISC-OCTA flow image was generated by masking the average image with the contrast image in step 3.

OCTA clearly represents an important technique for angiography due to its high resolution. Numerous OCTA systems have been successfully developed for humans, and OCTA algorithms have been reviewed and compared by Anqi Zhang *et al*., including optical microangiography (OMAG), speckle variance, Fourier domain mode locked (FDML), phase variance, split-spectrum amplitude-decorrelation angiography and correlation mapping^30^. The results show that OMAG and speckle variance may provide better visual results. In a previous study, we proposed the ISC-OCTA algorithm^31^. Through animal experiments and human retinal imaging experiments, the results showed that the ISC-OCTA algorithm can achieve better visual results and may be useful in the diagnosis of ophthalmic diseases. The schematic of the ISC-OCTA is illustrated in Fig. 7. N consecutive frames are averaged to obtain a mean image (*I*_*s*_(*x*, *y*, *z*)) as described in Eq. (1) per location, background noise is suppressed, and the intensity of blood flow signals is enhanced (step 1 in Fig. 7). A contrast image (*K*_*s*_(*x*, *y*, *z*)) is acquired by ISC processing for N consecutive frames as described in Eq. (2), which contains blood flow location information (step 2 in Fig. 7). Finally, the ISC-OCTA image (*I*_*flow*_ (*x*, *y*, *z*)) is generated by masking the averaged image with the contrast image as described in Eq. (3), the intensity of static tissue is suppressed, and the blood flow signals are preserved (step 3 in Fig. 7).

1 $${I}_{{\rm{s}}}(x,{y}_{j},z)=\frac{1}{N}\sum _{i=1}^{{\rm{N}}}I(x,{y}_{i},z)$$

2 $${K}_{s}(x,{y}_{j},z)=\frac{1}{N-1}\sum _{i=1}^{N-1}\frac{|I(x,{y}_{i+1}z)-I(x,{y}_{i},z)|}{I(x,{y}_{i+1},z)+I(x,{y}_{i},z)}$$

3 $${I}_{flow}(x,{y}_{j},z)=\frac{1}{N}\sum _{i=1}^{{\rm{N}}}I(x,{y}_{i},z)\ast \frac{1}{N-1}\sum _{i=1}^{N-1}\frac{|I(x,{y}_{i+1}z)-I(x,{y}_{i},z)|}{I(x,{y}_{i+1},z)+I(x,{y}_{i},z)}$$

### Establishment of left hindlimb ischemia animal models

Six-week-old female ICR mice were obtained from Vital River Laboratories (Beijing, China). The mice had free access to food and tap water ad libitum. All experimental procedures conducted in this study were performed in accordance with the relevant guidelines and regulations approved by the Institutional Animal Care and Use Committee at Peking Union Medical College. Sixteen female ICR mice were housed at 60 ± 10% humidity and a controlled temperature of 22 ± 2 °C with a light/dark cycle of 12 h. The animal model of hindlimb ischemia was established using previously described methods^19,32^. Briefly, animals were anesthetized by intraperitoneal administration of chloralic hydras (400 mg/kg, Xiya Reagent Co., Shandong Province, China), and both hindlimbs were shaved, depilated, and wiped with ethanol. A skin incision parallel to the left inguinal ligament was made to allow for the proper isolation, ligation, and excision of the femoral artery from its origin just above the inguinal ligament to its bifurcation at the origin of the saphenous and popliteal arteries. The incision was closed using 6-0 prolene sutures. After the operation, the mice were randomized into two groups. One group was treated with CuSO_4_ three times (40 μl, 4 μg/ml, once every two days), and the other group was treated with saline as a control. Each injection was performed smoothly over at least 15 s, and the needle was left in place for at least 10 s afterward to prevent back-leakage.

### Ischemic limb damage and weight assessment of the mice

At 0, 1, 3, 5 and 7 days after treatment, limb morphology was visually scored to evaluate the degree of ischemia-induced damage and body weight was also recorded. Five grades were used to measure the degree of ischemic limb injury according to previous literature^33^.

### Temperature ratio assessment of ischemic limbs

At 1, 3, 5 and 7 days after treatment, both hindlimb temperatures were evaluated by FLIR. The temperature ratio of ischemic to nonischemic hindlimbs was recorded. Evaluations were performed by an observer blinded to the treatment groups.

### Pathology and immunohistochemical assessment of ischemic limbs

The mice were euthanized on the 7th day after treatment. Tissue samples of ischemic muscles were separated and fixed in 4% paraformaldehyde for 24 h, and 7 μm frozen sections were prepared. The sections were stained with hematoxylin eosin for morphological analysis. Immunohistochemistry (IHC) staining of CD31 (1:400 dilution, Serotec, USA) was analyzed by counting 3 random high-power (magnification × 400) fields on an inverted light microscope and expressed as the number of CD31-positive cells per mm^2^. The area was measured with NIH image analysis software.

### OCTA assessment of ischemic limbs

For the quantitative assessment of the vasculature, the VAD was calculated within the entire image. Using the ISC-OCTA algorithm, we obtained a skin-free blood flow image. Due to the influence of the external factors during the image acquisition process, grayscale differences may have occurred in the different groups. To evaluate the microcirculatory VAD, we adjusted the gray level of all images to the same level and performed a morphological operation on the blood flow image. A binarized blood flow image was obtained by setting a threshold value, where “0” represents noise and “1” represents blood vessels. The binarized image included the blood vessel position information, and the pixels of the blood vessel were obtained by calculating the number of “1”. VAD is the blood vessel pixel values divided by the entire image pixel values, and the blood flow image was marked by the binarized image to obtain the final blood flow image. The microvasculature of the mouse left hindlimb was collected at three different timepoints (before and after femoral artery ligation and 7 days after treatment) when the skin needed to be incised.

### Statistics

Quantitative data are presented as the mean ± standard deviation (SD). Statistical significance was evaluated using the unpaired Student’s t-test and one-way analysis of variance (ANOVA). A P-value of 0.05 (*) indicated statistical significance.
